# Clinical Outcomes Among Patients With Metastatic Pancreatic Ductal Adenocarcinoma Treated With Liposomal Irinotecan

**DOI:** 10.3389/fonc.2021.678070

**Published:** 2021-07-15

**Authors:** Kenneth H. Yu, Andrew E. Hendifar, Olatunji B. Alese, Amber Draper, Maen Abdelrahim, Ethan Burns, Gazala Khan, Paul Cockrum, Rachel H. Bhak, Catherine Nguyen, Maral DerSarkissian, Mei Sheng Duh, Nathan Bahary

**Affiliations:** ^1^ Medicine/Gastrointestinal Oncology, Memorial Sloan Kettering Cancer Center and Weill Cornell Medicine, New York, NY, United States; ^2^ Hematology and Oncology, Samuel Oschin Comprehensive Cancer Center, Cedars-Sinai Medical Center, Los Angeles, CA, United States; ^3^ Department of Hematology and Medical Oncology, Emory Winship Cancer Institute, Atlanta, GA, United States; ^4^ Institute for Academic Medicine, Houston Methodist Cancer Center, Houston, TX, United States; ^5^ Department of Hematology-Oncology, Henry Ford Cancer Institute, Detroit, MI, United States; ^6^ Ipsen Biopharmaceuticals, Inc., Cambridge, MA, United States; ^7^ Analysis Group, Inc., Boston, MA, United States; ^8^ Department of Medical Oncology, University of Pittsburgh Medical Center, Pittsburgh, PA, United States

**Keywords:** metastasis, pancreatic ductal adenocarcinoma, cancer management, pancreatic cancer, liposomal irinotecan

## Abstract

**Background:**

The NAPOLI-1 trial demonstrated that liposomal irinotecan in combination with fluorouracil (5-FU) and leucovorin (LV) prolonged survival with a manageable safety profile in patients with metastatic pancreatic ductal adenocarcinoma (mPDAC) previously treated with gemcitabine-based therapy. Real-world data on clinical outcomes associated with liposomal irinotecan in NAPOLI-1-based regimens is needed to further substantiate this.

**Methods:**

This real-world, retrospective chart review study included patients with mPDAC who received NAPOLI-1-based regimens from six academic centers in the United States. Liposomal irinotecan initiation defined the index date. Overall survival (OS) and progression-free survival (PFS) were assessed with Kaplan-Meier methodology.

**Results:**

There were 374 patients evaluated; median age was 68 years, and 51% were female. Among 326 patients with baseline ECOG information, approximately 74% had ECOG score <2. Liposomal irinotecan was administered as a doublet with 5-FU in a NAPOLI-1-based regimen in the first line (1L; 16%), 2L (42%), and 3L+ (42%) of the metastatic setting. For patients treated in 1L, 2L, and 3L+, median [95% confidence interval (CI)] OS was 8.0 [5.1, 11.2], 7.3 [5.3, 8.8], and 4.6 [4.0, 5.7] months, and median [95% CI] PFS was 4.2 [2.2, 6.6], 3.0 [2.6, 3.7], and 2.0 [1.7, 2.2] months, respectively.

**Conclusions:**

Patients in a real-world setting treated with NAPOLI-1-based liposomal irinotecan doublet regimens at academic centers were older with poorer performance status compared to trial patients yet had similar outcomes and efficacy. Furthermore, liposomal irinotecan was frequently used in the 3L+ setting where no treatment has been approved and provided clinical benefit.

## Introduction

Despite recent diagnostic and therapeutic advances, pancreatic cancer remains an aggressive and difficult to treat malignancy. Although it only comprises 3% of new cancer diagnoses, it is projected to be the second leading cause of cancer-related mortality by 2030 (1). Due to an absence of effective screening tools, pancreatic cancer is frequently diagnosed when locally advanced or widely metastatic. Delayed diagnosis contributes to treatment challenges as surgical resection is the only means to curative treatment and is a factor in the poor 5-year survival rate ranging from 3-8% (2).

In October 2015, the Food and Drug Administration (FDA) approved liposomal irinotecan in combination with 5-fluorouracil (5-FU) and leucovorin (LV) for the treatment of metastatic pancreatic ductal adenocarcinoma (mPDAC) in patients that had previously progressed on gemcitabine-based chemotherapy following the results of the pivotal NAPOLI-1 trial. The NAPOLI-1 trial evaluated liposomal irinotecan in combination with 5-FU/LV compared to treatment with 5-FU/LV alone in patients with mPDAC previously treated with gemcitabine-based therapy (3). The results indicated that treatment with liposomal irinotecan in combination with 5-FU/LV compared to 5-FU/LV alone significantly prolonged the median overall survival (OS) (6.1 months *vs.* 4.2 months; hazard ratio [HR]: 0.67; p: 0.012) and median progression-free survival (PFS) (3.1 months *vs.* 1.5 months; HR: 0.56; p: 0.0001) in patients with mPDAC. Liposomal encapsulation prolongs the duration of circulating irinotecan prior to conversion to its active metabolite SN-38, thereby protecting irinotecan from hydrolysis and rapid metabolic conversion (4, 5). Liposomal irinotecan in combination with 5-FU/LV is the only category 1 treatment recommended by the National Comprehensive Cancer Network (NCCN) for patients with mPDAC after disease progression following gemcitabine-based therapy (6).

While the NAPOLI-1 trial results have expanded the treatment options for mPDAC, there are limited real-world data evaluating the use and outcomes of treatment with liposomal irinotecan. A single institution study conducted at Memorial Sloan Kettering Cancer Center (MSKCC) in 2017 assessed similar treatment outcomes among patients with mPDAC treated with liposomal irinotecan and reported similar results (median OS 5.3 months and median PFS 2.9 months) to NAPOLI-1 (7). To expand on the aforementioned study’s findings, this real-world study incorporated patients from five additional cancer centers across the United States (US) in order to assess real-world outcomes, treatment patterns, and adverse events (AE) in patients with mPDAC treated with liposomal irinotecan in a NAPOLI-1-based doublet regimen.

## Methods

### Study Design and Study Population

This was a non-interventional, retrospective, multi-center chart review study that was conducted using data from six academic cancer centers across the US. Participating centers included MSKCC, Cedars-Sinai Medical Center, Emory Winship Cancer Institute, Houston Methodist Cancer Center, Henry Ford Cancer Institute, and University of Pittsburgh Medical Center. Eligible patients were treated with liposomal irinotecan in a doublet with 5-FU between 2015 and 2020 and were diagnosed with mPDAC at any time before liposomal irinotecan initiation. Following IRB approval at each participating center, designated abstractors collected patient demographics, clinical characteristics and outcomes, and treatments from patient medical charts and electronic medical records using a standardized electronic case report form (eCRF). This study was conducted in two phases: a pilot phase and a full-launch phase. During the pilot phase, the eCRF was prepared and tested at one center using data from ten patient charts. Reviewers ensured that the eCRF accurately captured all relevant information and that data was collected as efficiently as possible. Based on feedback from the pilot phase, the eCRF was then updated and finalized for the full launch phase where data collection began at all centers.

**Figure 1:**
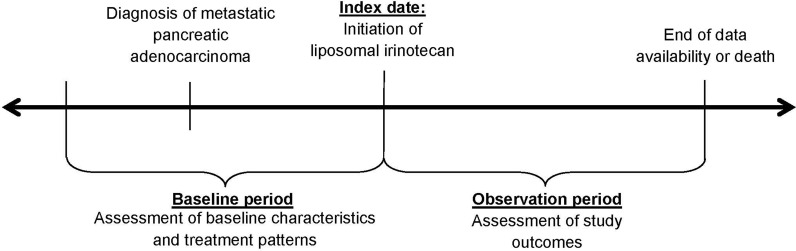
Study Design.

Patient data were collected during the baseline and observation periods before and after the initiation of liposomal irinotecan, the index date. The baseline period captured data available prior to the index date (until the date of initial pancreatic cancer diagnosis if available). Study outcomes were assessed during the observation period, defined as the period from the index date to the end of data availability or death (**Figure 1**).

### Study Variables and Outcomes

Patient demographics (e.g., age, region) and clinical characteristics (e.g., Eastern Cooperative Oncology Group [ECOG] performance status) were assessed during the baseline period or at index date. For patients with missing baseline ECOG performance status, their Karnofsky Performance Status (KPS) scores were converted to ECOG status.

Study outcomes included treatment patterns, real-world effectiveness (i.e., OS and PFS), and grade 3 or 4 AEs. Duration of liposomal irinotecan treatment was defined as the time from index date to discontinuation. Treatment patterns of therapies received in the metastatic setting prior to liposomal irinotecan were examined. Two definitions of OS were used: (1) OS from mPDAC diagnosis to death, and (2) OS from index date to death. PFS was calculated from index date to the earliest of disease progression or death. Clinically meaningful symptom-related grade 3 or 4 AEs were reported, based on the Common Terminology Criteria for Adverse Events (8).

### Statistical Analyses

Summary statistics were presented as means, standard deviations (SDs), and medians for continuous variables or frequencies and proportions for categorical variables. Time to event analyses were conducted using Kaplan-Meier methodology. For OS, patients were censored at the end of data availability, and for PFS, patients were censored at liposomal irinotecan discontinuation or end of data availability. Time to event analyses were stratified by the line of therapy in the metastatic setting that patients received liposomal irinotecan [i.e., first-line (1L), second-line (2L), or third-line or later (3L+)].

The association between baseline characteristics and effectiveness outcomes [i.e., OS (from index), PFS] was analyzed using a Cox proportional hazards model. HRs, 95% confidence intervals (CIs), and p-values were reported. P-values from all statistical tests were reported based on an alpha level of 0.05. All analyses were performed using SAS version 9.4 (SAS Institute, Inc., Cary, NC).

## Results

### Baseline Characteristics

374 patients met the study eligibility criteria and were included in this study (**Table 1**). The mean ± SD [median] age at index date was 67.8 ± 9.4 [68.6] years, and 51.3% of patients were female. Most patients were white (71.7%), from the Northeast (57.0%), and initiated liposomal irinotecan treatment in 2018 (32.6%) or 2019 (24.9%). The majority of patients (50.8%) had stage IV pancreatic cancer at initial diagnosis. Patients were treated with liposomal irinotecan in the 1L (17%), 2L (42%), or 3L+ (42%) of the metastatic setting. Among patients treated with liposomal irinotecan in 3L+, 57.0% were treated with a 5-FU-based regimen and 41.7% were treated with a gemcitabine-based regimen in 1L setting; 53.2% were treated with a gemcitabine-based regimen and 34.6% were treated with a 5-FU-based regimen in 2L setting. Among 326 patients with available performance status information, approximately 74% of patients had a baseline ECOG performance status of 0-1. Diabetes without end-organ damage was the most common comorbid condition, present in 25.4% of patients.

**Table 1:** Baseline demographic and clinical characteristics.

	N = 374
***Demographic Characteristics***	** **
**Age (years) at index, mean ± SD [median]**	67.8 ± 9.4 [68.6]
**Female, n (%)**	192 (51.3)
**Race/Ethnicity, n (%)**	
White	268 (71.7)
Black/African-American	48 (12.8)
Asian/Pacific Islander	26 (7.0)
Hispanic/Latino	13 (3.5)
Native American/American Indian	1 (0.3)
Unknown	18 (4.8)
**Geographic location, n (%)**	
Northeast	213 (57.0)
South	78 (20.9)
West	57 (15.2)
Midwest	26 (7.0)
**Year of index, n (%)**	
2015	1 (0.3)
2016	63 (16.8)
2017	82 (21.9)
2018	122 (32.6)
2019	93 (24.9)
2020	13 (3.5)
***Clinical Characteristics***	
**Time from mPDAC diagnosis to index (months),** **mean ± SD [median]**	10.9 ± 9.9 [8.4]
**Cancer stage at first diagnosis of PDAC, n (%)**	
I A	4 (1.1)
I B	6 (1.6)
II A	22 (5.9)
II B	52 (13.9)
III	72 (19.3)
IV	190 (50.8)
Unknown	28 (7.5)
**Liposomal irinotecan line of therapy, n (%)**	
1L	62 (16.6)
2L	156 (41.7)
3L+	156 (41.7)
**Primary tumor location in pancreas, n (%)**	
Head	207 (55.3)
Body	65 (17.4)
Tail	62 (16.6)
Body and tail	32 (8.6)
Neck	2 (0.5)
Neck and body	1 (0.3)
Unknown	5 (1.3)
**Metastatic sites, n (%)**^**a**^	
Liver	258 (69.0)
Lung	87 (23.3)
Peritoneum	79 (21.1)
Distant lymph nodes	51 (13.6)
Bone	16 (4.3)
Brain	3 (0.8)
Other^b^	25 (6.7)
**Number of metastatic sites, n (%)**	
1	280 (74.9)
2	57 (15.2)
3 or more	37 (9.9)
**ECOG performance score, n (%)**	
0	32 (8.6)
1	211 (56.4)
2	73 (19.5)
3	8 (2.1)
4	2 (0.5)
Unknown	48 (12.8)
**Selected comorbidities, n (%)**^**a**^^**,**^^**c**^	
Diabetes without end-organ damage	95 (25.4)
Peripheral vascular disease	16 (4.3)
Diabetes with end-organ damage	16 (4.3)
Chronic obstructive pulmonary disease	12 (3.2)
Moderate or severe renal disease	12 (3.2)
Congestive heart failure	10 (2.7)
Cerebrovascular disease	9 (2.4)

ECOG, eastern cooperative oncology group; mPDAC, metastatic pancreatic ductal adenocarcinoma; PDAC, pancreatic ductal adenocarcinoma, SD, standard deviation.

a Patients may have ≥1 value reported. Therefore, the sum of the percentages may be greater than 100%.

b Other metastatic sites included abdominal wall, adrenal glands, ascites, chest wall, diaphragm, gastric, gluteus muscle, kidney, ovary, pelvis, right adnexa, serosa, spleen, and thyroid.

c The listed comorbid conditions belong to the Charlson Comorbidity Index.

**Table 2** provides information on 312 patients who received treatment in the metastatic setting prior to liposomal irinotecan. Gemcitabine-based therapy was received among 93.9% of patients. **Table 3** details liposomal irinotecan treatment characteristics. The overall median [95% CI] treatment duration of liposomal irinotecan was 1.6 [1.4, 1.9] months and was 2.8 [1.4, 5.6], 2.1 [1.6, 2.8], and 1.4 [1.3, 1.6] months for patients treated with liposomal irinotecan in 1L, 2L, and 3L+, respectively. Twenty three patients had treatment duration of liposomal irinotecan longer than 12 months, 82.6% of whom were treated with liposomal irinotecan in 1L or 2L. In addition, 9 patients had treatment duration of liposomal irinotecan longer than 18 months, and 5 patients had treatment duration of liposomal irinotecan longer than 24 months. Among 2L patients (n=156), 1.3% had prior irinotecan, and among 3L patients (n=156), 57.7% had prior irinotecan in the metastatic setting. Among 367 patients with dosing information, 29.4% patients had dose reduction at any time during liposomal irinotecan treatment. 7.0% of patients received granulocyte colony stimulating factor (GCSF) with their first administration of liposomal irinotecan.

**Table 2:** Treatment patterns in the metastatic setting prior to liposomal irinotecan-based treatment.

	N = 374
**Duration of metastatic treatments prior to liposomal irinotecan (months), mean ± SD [median]**^**a**^	9.4 ± 8.4 [7.0]
**Treatment regimens prior to liposomal irinotecan, n (%)** ^**b**^^**,**^^**c**^	*312*
** Gemcitabine (alone or in combination)**	293 (93.9)
Gem + nab-P	226 (77.1)
Gemcitabine	48 (16.4)
Gem + nab-P + cisplatin	9 (3.1)
Gem + cisplatin	7 (2.4)
Gem + paclitaxel	5 (1.7)
** 5-FU (alone or in combination)**	122 (39.1)
FOLFIRINOX	83 (68.0)
FOLFOX	37 (30.3)
FOLFIRI	32 (26.2)
5-FU/(LV)	15 (12.3)
**Any irinotecan (alone or in combination), n (%)**	92 (29.5)
**Received irinotecan (alone or in combination) in the line of therapy prior to the first administration of liposomal irinotecan, n (%)**	12 (3.8)

5-FU, 5-fluorouracil; FOLFIRI, 5-FU + leucovorin + irinotecan; FOLFIRINOX, 5-FU + leucovorin + irinotecan + oxaliplatin; FOLFOX, 5-FU + leucovorin + oxaliplatin; Gem, gemcitabine; LV, leucovorin or levoleucovorin; mPDAC, metastatic pancreatic ductal adenocarcinoma; nab-P, nab-paclitaxel; PEGPH20, PEGylated recombinant human hyaluronidase; SD, standard deviation.

a Treatment duration was defined as cumulative duration of any treatment regimen. It was calculated among patients who had any treatment prior to liposomal irinotecan initiation.

b Patients may have ≥ 1 value reported. Therefore, the sum of the percentages may be greater than 100%.

c Treatment regimens were categorized by grouping together treatments that were initiated within 30 days of each other.

**Table 3:** Liposomal irinotecan treatment characteristics in the metastatic setting.

	N = 374
**Duration of liposomal irinotecan (months), median [95% CI]**	
All patients	1.6 [1.4, 1.9]
1L (n=62)	2.8 [1.4, 5.6]
2L (n=156)	2.1 [1.6, 2.8]
3L+ (n=156)	1.4 [1.3, 1.6]
**Prior irinotecan in the metastatic setting, n (%)**	
All patients	92 (29.5)
2L (n=156)	2 (1.3)
3L+ (n=156)	90 (57.7)
**Liposomal irinotecan dosage, n (%)**	
Patients with dose information available	*367*
Patients with dose modifications, n (%)	116 (31.6)
Patients with dose reduction, n (%)	108 (29.4)
**Treatments concomitant with liposomal irinotecan, n (%)**^**a**^	
5-FU/(LV)	358 (95.7)
Other	4 (1.1)
GCSF	80 (21.4)
Pegfilgrastim	75 (93.8)
Filgrastim	14 (17.5)
Tbo-filgrastim	3 (3.8)
Filgrastim-sndz	1 (1.3)
**GCSF with first administration of liposomal irinotecan**^**b**^	26 (7.0)
**Any 5-FU (alone or in combination)**	374 (100.0)

5-FU, 5-fluorouracil; CI, confidence interval; GCSF, granulocyte colony stimulating factor; LV, leucovorin; m, meter; mg, milligram; mPDAC, metastatic pancreatic ductal adenocarcinoma; SD, standard deviation.

a Patients may have ≥ 1 value reported. Therefore, the sum of the percentages may be greater than 100%.

b Patients that had GCSF administered between one and four days after index date are displayed, based on the NCCN Guidelines on Hematopoietic Growth Factors, Version 2.2020.

### Real-World Effectiveness

**Figure 2:**
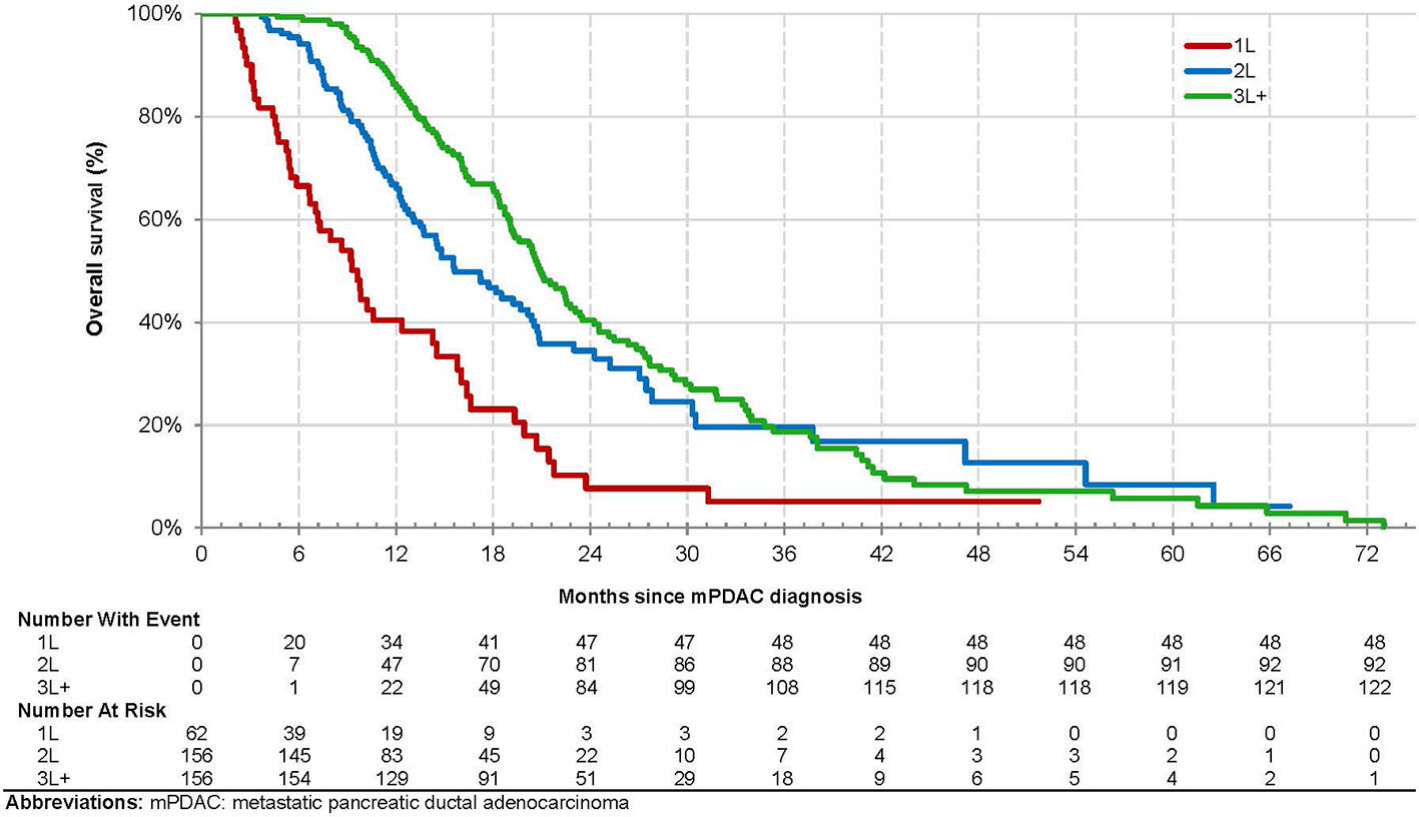
Kaplan-Meier Analysis of Overall Survival from mPDAC Diagnosis among Patients with mPDAC Treated with Liposomal Irinotecan in a Doublet with 5-Fluorouracil.

**Figure 3:**
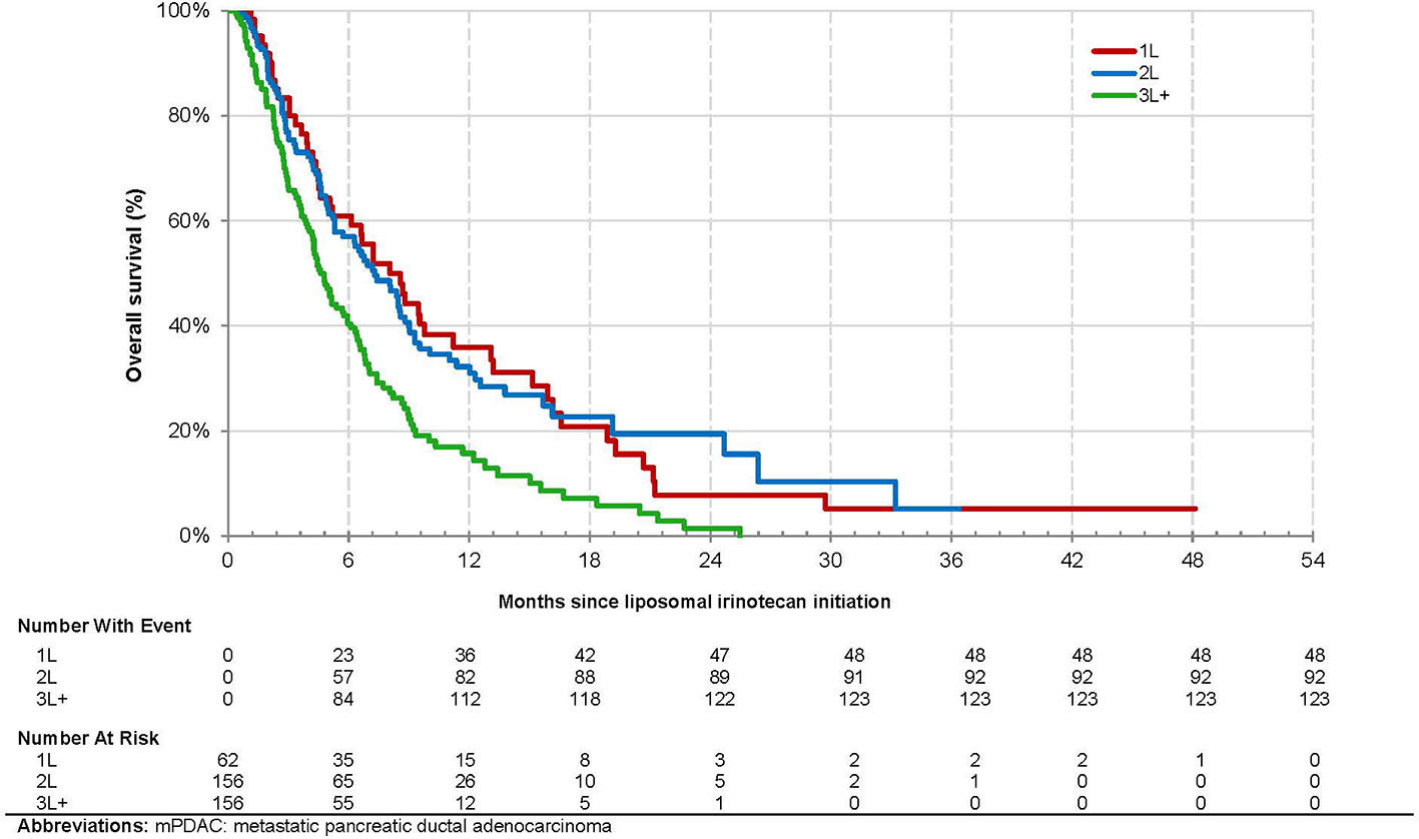
Kaplan-Meier Analysis of Overall Survival from Initiation of Treatment with Liposomal Irinotecan among Patients with mPDAC Treated with Liposomal Irinotecan in a Doublet with 5-Fluorouracil.

**Figure 4:**
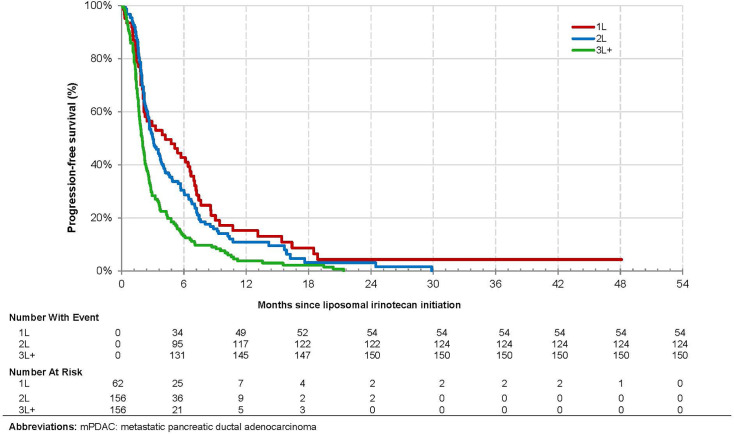
Kaplan-Meier Analysis of Progression Free Survival among Patients with mPDAC Treated with Liposomal Irinotecan in a Doublet with 5-Fluorouracil.

Overall, 263 (70.3%) patients died, and 328 (87.7%) patients experienced disease progression or died over the observation period. The overall median (95% CI) OS from mPDAC diagnosis was 18.4 [16.1, 19.9] months and 9.6 (6.7, 14.3), 15.6 (13.5, 20.4), and 20.9 [19.1, 23.4] for patients treated with liposomal irinotecan in 1L, 2L, and 3L+, respectively (**Figure 2**). The overall median [95% CI] OS from the index date was 6.1 (5.1, 6.8) months and 8.0 (5.1, 11.2), 7.3 (5.3, 8.8), and 4.6 (4.0, 5.7) months for patients treated with liposomal irinotecan in 1L, 2L, and 3L+, respectively (**Figure 3**). The overall median [95% CI] PFS was 2.5 (2.2, 2.8) months and 4.2 (2.2, 6.6), 3.0 (2.6, 3.7), and 2.0 (1.7, 2.2) for patients treated with liposomal irinotecan in 1L, 2L, and 3L+, respectively (**Figure 4**).

The results of the multivariate Cox model analyzing the association between the baseline characteristics and effectiveness outcomes are presented in **Table 4**. In the model examining OS, patients treated with liposomal irinotecan in 3L+ *vs.* 2L had a significantly higher risk of death [HR: 1.90 (1.38, 2.63), p: < 0.001]. Patients with liver metastases [1.59 (1.19, 2.11), p: 0.002], brain metastases [6.53 (1.83, 23.29), p: 0.004], and congestive heart failure (3.01 [1.45, 6.26], p: 0.003) also had a significantly higher risk of death.

**Table 4:** Associations between Baseline Characteristics and Effectiveness Outcomes of Liposomal Irinotecan - Multivariate Analysis.

	OS			PFS		
	N = 374			N = 374		
	HR	95% CI	P-value^3^	HR	95% CI	P-value^3^
**Age at index date**	1.00	(0.98, 1.01)	0.792	0.99	(0.98, 1.01)	0.248
**Male (ref: female)**	1.18	(0.92, 1.53)	0.189	1.05	(0.84, 1.32)	0.669
**Race/Ethnicity (ref: white)**						
Black/African-American	1.14	(0.72, 1.82)	0.569	0.92	(0.62, 1.36)	0.673
Hispanic/Latino	0.88	(0.43, 1.81)	0.736	1.03	(0.52, 2.05)	0.940
Asian/Pacific Islander and Native American/American Indian	0.80	(0.47, 1.37)	0.413	0.79	(0.50, 1.25)	0.316
Unknown	0.79	(0.45, 1.40)	0.424	0.63	(0.37, 1.07)	0.088
**Geographic Location** **(ref: northeast)**						
Midwest	0.79	(0.47, 1.35)	0.394	1.00	(0.62, 1.61)	0.999
South	0.60	(0.38, 0.94)	0.026*	0.98	(0.68, 1.42)	0.923
West	0.46	(0.29, 0.73)	<0.001*	0.65	(0.44, 0.96)	0.030*
**Time from mPDAC diagnosis to index (months)**	0.98	(0.96, 1.00)	0.013*	0.97	(0.96, 0.99)	0.001*
**Cancer stage at first diagnosis of PDAC (ref: metastatic)**						
Non-metastatic	0.84	(0.60, 1.18)	0.313	0.91	(0.67, 1.23)	0.525
Unknown	0.77	(0.41, 1.43)	0.406	0.80	(0.49, 1.31)	0.372
**Line of therapy for liposomal irinotecan (ref: 2L)**						
1L	0.80	(0.51, 1.27)	0.349	0.78	(0.52, 1.18)	0.246
3L+	1.90	(1.38, 2.63)	<0.001*	1.99	(1.49, 2.65)	<0.001*
**Primary tumor location in pancreas (ref: head)**						
Body	0.70	(0.49, 1.00)	0.048*	0.62	(0.45, 0.86)	0.004*
Body and tail	0.83	(0.53, 1.33)	0.443	0.72	(0.47, 1.10)	0.125
Neck or neck and body	0.81	(0.10, 6.23)	0.838	0.27	(0.04, 2.00)	0.199
Tail	1.01	(0.70, 1.45)	0.970	1.22	(0.89, 1.68)	0.222
Unknown	0.46	(0.06, 3.41)	0.448	0.65	(0.20, 2.15)	0.479
**Metastatic sites**						
Liver	1.59	(1.19, 2.11)	0.002*	1.63	(1.26, 2.10)	<0.001*
Brain	6.53	(1.83, 23.29)	0.004*	2.53	(0.73, 8.74)	0.144
**Number of metastatic sites** **(ref: 1)**						
2	0.99	(0.67, 1.45)	0.941	1.18	(0.85, 1.64)	0.316
3 or more	1.09	(0.65, 1.81)	0.744	1.05	(0.68, 1.62)	0.842
**ECOG (ref: < 2)**						
≥ 2	1.21	(0.87, 1.69)	0.254	1.01	(0.75, 1.34)	0.970
Unknown	1.09	(0.72, 1.65)	0.699	1.12	(0.77, 1.62)	0.555
**Selected comorbidities**						
Congestive heart failure	3.01	(1.45, 6.26)	0.003*	2.53	(1.29, 4.99)	0.007*

CI, confidence interval; ECOG, Eastern Cooperative Oncology Group; HR, hazard ratio; mPDAC, metastatic pancreatic ductal adenocarcinoma; OS, overall survival; PDAC, pancreatic ductal adenocarcinoma; PFS, progression free survival. *indicates p-value <0.05.

In the model examining PFS, patients treated with liposomal irinotecan in 3L+ *vs.* 2L also had a significantly higher risk of tumor progression/death [HR (95% CI): 1.99 (1.49, 2.65), p: < 0.001]. Consistent with OS, patients with liver metastases [1.63 (1.26, 2.10), p: < 0.001] and congestive heart failure [2.53 (1.29, 4.99), p: 0.007] had significantly higher risk of tumor progression/death.

### Grade 3 or 4 Adverse Events

The most common grade 3 or 4 symptom-related AEs were fatigue/asthenia (4.0%), diarrhea (3.2%), and vomiting (1.6%). The most common grade 3 or 4 laboratory abnormalities were anemia (21.1%), lymphopenia (12.6%), and neutropenia (7.8%) (**Table 5**).

**Table 5:** Grade 3 or 4 symptom-related adverse events and laboratory abnormalities.

	N = 374
**Symptom-related adverse events, n (%)**	
Fatigue/asthenia	15 (4.0)
Diarrhea	12 (3.2)
Vomiting	7 (1.9)
Nausea	6 (1.6)
**Laboratory abnormalities, n (%)**	
Anemia	79 (21.1)
Lymphopenia	47 (12.6)
Neutropenia	29 (7.8)

## Discussion

From six academic centers, 374 patients with mPDAC treated with liposomal irinotecan in a doublet with 5-FU were examined. The results of this retrospective, observational chart review study indicate that patients with mPDAC treated with liposomal irinotecan in the real-world setting compared to the pivotal phase 3 clinical trial (NAPOLI-1) (3) were older (median age: 69 *vs.* 63 years), had poorer ECOG performance status (ECOG <2: 74% *vs.* 91%), and had received more lines of therapy prior to liposomal irinotecan (2+ prior lines of therapy: 42% *vs.* 34%). Nonetheless, the median OS in this study was identical to data reported in the NAPOLI-1 trial (median OS: 6.1 months) (3). This study’s results are also consistent with those from a real-world Flatiron study on patients with mPDAC treated with liposomal irinotecan in the community setting and reinforce the conclusions from the Glassman et al. study (7, 9).

Overall, liposomal irinotecan was effective for treatment of mPDAC in the study population, particularly among those receiving it in earlier lines of therapy. Patients treated with liposomal irinotecan in 1L, 2L, and 3L+ had median OS of 8.0 months, 7.3 months, and 4.6 months, respectively. This trend was similarly reported in the Flatiron study where patients treated with liposomal irinotecan in 1L, 2L, and 3L+ had median OS of 6.9, 5.4, and 4.0 months, respectively (9). Similarly, Glassman et al. reported an overall median OS of 5.3 months and longer OS in patients receiving liposomal irinotecan in earlier lines of therapy (7). Thus, the observed survival trends among patients treated with liposomal irinotecan in 1L, 2L and 3L+ are similar to other real world studies. In addition, this study found in adjusted analyses that patients treated with liposomal irinotecan in 3L+ *vs.* 2L had significantly higher risk of death. This study also reported that patients treated with liposomal irinotecan in earlier *vs.* later lines of therapy had better PFS. These results demonstrate the clinical benefit of being treated with liposomal irinotecan in earlier *vs.* later lines of therapy, which may be attributed to common resistance mechanisms, more severe disease, and worse prognosis/performance status in patients with each subsequent line of therapy.

To complement the OS and PFS benefit conferred by liposomal irinotecan, this study also described the safety profile of liposomal irinotecan in patients that were older and had poorer baseline performance status when compared to the pivotal NAPOLI-1 trial. It is noted that due to the real-world nature of this study, AEs may not have been recorded as often as AEs in clinical trials where patients are monitored more closely. Compared to other real-world settings, this study had a lower proportion of patients with grade 3 or 4 neutropenia than that previously reported in the community setting (8% *vs.* 11%) (9), and a slightly higher proportion of patients with various grade 3 or 4 AEs than in Glassman et al. (7). Overall, this study further supports the known safety profile and use of liposomal irinotecan.

Findings from this study may inform treatment recommendations for patients with mPDAC. Currently, the NCCN recommends liposomal irinotecan as a 2L therapy for patients with mPDAC and good performance status following treatment with a gemcitabine-based regimen (10). They do not list any recommended treatments for 1L or 3L+ for patients with mPDAC. This real-world study indicates promising survival among patients treated with liposomal irinotecan in 3L+, and may support using liposomal irinotecan in later lines of therapy when other treatment options are not available. Additionally, liposomal irinotecan was used as 1L therapy in a number of patients in this study despite not being indicated for front-line use; this could be due to several reasons: failure of adjuvant gemcitabine-based therapy, possible neuropathy, or patient/provider preferences based on toxicity profiles. This study therefore describes real-world use of liposomal irinotecan in circumstances where patients may not exactly fit clinical trial entry criteria.

There are several limitations to consider when interpreting findings from this study. Due to the non-randomized, retrospective nature of the study, residual confounding may impact the associations and conclusions identified. Specifically, residual confounding may have remained for the comparative analyses by line of therapy even after adjustment (e.g., patients receiving liposomal irinotecan in 3L may have been sicker than those receiving liposomal irinotecan in 1L). The results reported are based on data collected at academic cancer centers and may not be generalizable to patients with mPDAC treated in other settings. Real-world evidence from medical charts is also limited by the availability of clinical data reported in the medical chart, although quality assurance procedures and data checks served to maximize data integrity. In addition, in analyses where less common conditions such as brain metastases or certain comorbidities are examined, the smaller number of patients with these conditions could limit the ability for robust conclusions. However, the findings from this study are corroborated by existing literature that report poorer prognostic outcomes among patients with pancreatic cancer who have brain metastases or comorbidities (11, 12). Furthermore, this study overall included a large number of patients to describe the current treatment landscape for mPDAC and evaluate NAPOLI-1-based liposomal irinotecan doublet regimens in the real-world setting. Baseline information on AEs were not collected, so it is unclear if the AE data reported are treatment emergent (i.e., associated with liposomal irinotecan). The assessments of disease progression and AE grading in real-world settings may be based on heterogeneous criteria and assessment schedules across subjects and centers. For example, PFS may be overestimated if the patient’s visit and evaluation of progression was recorded in the patient’s chart later than the actual date of progression itself.

Poor prognosis among patients with mPDAC necessitates continuous research on efficacious and better tolerated treatments to improve patient outcomes. This real-world study found that patients treated with liposomal irinotecan were older, sicker, and had more lines of therapy prior to liposomal irinotecan than those in the NAPOLI-1 registrational trial; however, real-world effectiveness was similar. Furthermore, patients were treated with liposomal irinotecan in 3L+, a setting with no currently approved options, and demonstrated clinical benefit.

## Data Availability Statement

The original contributions presented in the study are included in the article/supplementary files. Further inquiries can be directed to the corresponding author.

## Ethics Statement

Prior to commencing this study, each academic center that participated in this study received institutional review board (IRB) approval at their respective institution or received approval through a centralized IRB. Specific IRB approval details are listed below:

- Memorial Sloan Kettering Cancer Center IRB approval: #17-302
- Cedars-Sinai Medical Center IRB approval: Pro00057619
- Emory University IRB approval: #00111751
- Houston Methodist Research Institute IRB approval: Pro00023019
- Henry Ford Health System IRB approval: #13477
- Analysis Group - New England IRB Approved under Exempt Category: #1-8654-1
- University of Pittsburgh Medical Center - New England IRB Approved under Exempt Category: #1-8854-1

This study was implemented and reported in accordance with the ethical principles set forth in the Declaration of Helsinki. Written informed consent for participation was not required for this study in accordance with the national legislation and the institutional requirements.

## Author Contributions

KHY, AEH, OBA, AD, MA, EB, GK, and NB: Conceptualization, data curation, methodology, writing–review and editing. PC, Conceptualization, funding acquisition, writing-review and editing. MSD, MD, RHB, and CN: Conceptualization, formal analysis, methodology, project administration, writing - original draft. All authors contributed to the article and approved the submitted version.

## Funding

This study was funded by Ipsen Biopharmaceuticals, Inc.

## Conflict of Interest

KHY: Research Funding (BMS, Ipsen, Halozyme); Advisory Board (Ipsen). AEH: Consulting or Advisory Role (Novartis, Ipsen, Perthera, Celgene, Abbvie); Research Funding (Ipsen); Travel, Accommodations, Expenses (Halozyme). OBA: Research Funding (Taiho Oncology, Ipsen Pharmaceuticals, GSK, Bristol Myers Squibb, PCI Biotech AS, Calithera Biosciences, Inc., SynCore Biotechnology Co., Ltd., Corcept, Mabspace Biosciences); Consulting/Advisory Role (Exelixis, Conjupro BioTherapeutics, R-Pharm US LLC, Ipsen Pharmaceuticals, Natera, Taiho, Pfizer, QED therapeutics). MA: Advisory Board and Speaker (Ipsen). NB: Consultant (AstraZeneca, Exelixis, BMS, Thermo Fisher). PC is an employee of Ipsen Biopharmaceuticals, Inc. and owns stock/stock options. MSD, MD, RHB, and CN are employees of Analysis Group Inc., which has received consultancy fees from Ipsen Biopharmaceuticals, Inc.

The remaining authors declare that the research was conducted in the absence of any commercial or financial relationships that could be construed as a potential conflict of interest.
